# Correlations of SDF-1ɑ and XRCC1 gene polymorphisms with the risk of renal cancer development and bioinformatics studies of SDF-1α and XRCC1 and the prognosis of renal cancer

**DOI:** 10.1038/s41598-024-53808-4

**Published:** 2024-02-09

**Authors:** Wenjing Zhang, Yubo Su, Genquan Yue, Lingyan Zhao, Hailing Li, Min Jia, Yuqi Wang, Dongyang Liu, Haisheng Wang, Yumin Gao

**Affiliations:** 1 School of Public Health, Inner Mongolia Medical University, Hohhot, China; 2 Department of Urology, Affiliated Hospital of Inner Mongolia Medical University, Hohhot, China; 3 Key Laboratory of Molecular Epidemiology of Chronic Diseases, Inner Mongolia Medical University, Hohhot, China; 4 Department of Biochemistry and Molecular Biology, School of Basic Medicine, Inner Mongolia Medical University, Hohhot, China

**Keywords:** SDF-1α gene, XRCC1 gene, SNP, RCC, Bioinformatics analysis, Urological cancer, Renal cancer

## Abstract

To study the relationships between stromal cell-derived factor-1 (SDF-1ɑ) and renal cell carcinoma (RCC) susceptibility and the presence of single nucleotide polymorphisms in the human X-ray cross-complementary repair gene (XRCC1). Compare SDF-1 based on RCC related data in the TCGA database α, The expression difference of XRCC1 between RCC tissue and normal tissue; Collect 166 newly diagnosed RCC cases and 166 healthy individuals who underwent physical examinations during the same period, and detect genotype using iMLDR method. The results The rs1801157 locus (C:T) of the SDF-1α gene was not significantly associated with the pathohistological type, the rs1799782 locus (G:A) of the XRCC1 gene was associated with the pathohistological type of RCC, and there were interactions between rs1799782 and smoking, alcohol consumption, pesticide exposure, hair dye, and urine holding. The rs1799782 locus of the XRCC1 gene may be a key factor in the pathogenesis and pathological development of RCC. High SDF-1ɑ expression is a protective factor for the overall survival of patients with RCC, and SDF-1ɑ and XRCC1 may be important for the treatment of RCC.

## Introduction

Renal cell carcinoma (RCC) is the most common type of genitourinary cancer. It has a mortality rate of 30–40% and is more common in men than^1^ in women. The incidence of renal cancer varies between countries and regions of the world, with developed countries having a higher incidence than developing countries^2^. The incidence of kidney cancer varies in different countries and regions of the world. In recent years, the incidence rate of kidney cancer has shown a rising trend year by year, and the age of onset of the disease is mostly concentrated in the age of 50–70 years old, and with the popularization of health checkups, there is a tendency for the age of onset of kidney cancer patients to advance^3^. At present, surgery is the main treatment measure for kidney cancer patients, kidney cancer patients are not sensitive to radiotherapy or chemotherapy, and there is a lack of effective treatment means for patients with advanced kidney cancer who cannot be resected^4^. Patients with advanced kidney cancer who cannot undergo resection lack effective treatment. Epidemiologic investigations have shown that approximately 3% of RCC patients have a family history with an autosomal dominant pattern, suggesting that genetic factors play an important role in RCC susceptibility^1^. Among the various genetic factors, the role of gene polymorphisms is gradually being recognized. Tong^5^, in a meta-analysis, reported that the SDF-1ɑ G801A gene polymorphism strongly increased cancer risk in Asians. Hirata et al.^6^ also revealed an association between the XRCC1 399Gln polymorphism and RCC risk.

Based on the above evidence, this study used a case‒control method to analyze the relationship between SDF-1ɑ(also known as the chemokine CXCL12) or XRCC1 and RCC and further analyzed the expression of the SDF-1ɑ and XRCC1 genes in RCC via bioinformatics methods, which can provide new clues and references for the active prevention and treatment of RCC.

## Objects and methods

### Bioinformatics methods

The Cancer Genome Atlas (TCGA) database^7^ was used to download SDF-1ɑ and XRCC1 gene expression data and corresponding clinical information from renal cancer patients; compare the expression levels of the target genes in tumor tissues and normal tissues; and perform Cox regression to analyze the relationship between SDF-1ɑ and overall survival. *P* < 0.05 indicated that the difference was statistically significant. Gene Ontology (GO) functional enrichment analysis^8^ and gene set enrichment analysis (GSEA)^9^ were performed on the SDF-1ɑ gene to determine the signaling pathway through which SDF-1ɑ may participate in and explore the possible mechanism of the SDF-1ɑ gene's involvement in the development and development of renal cancer.

XRCC1 and SDF-1ɑ(CXCL12) expression data were obtained from 542 cancer tissue samples and 72 paracancerous tissue samples using The Cancer Genome Atlas (TCGA) transcriptome RNA-seq data and normal tissue data were simultaneously log2 transformed to match the differential expression information between tumor and normal tissues. Differences in SDF-1ɑ expression were explored using the R package “limma”, and *p* < 0.05 was considered to indicate statistical significance.

After downloading and organizing the differential expression data for the SDF-1ɑ gene from the TCGA database, we explored the potential functional enrichment of SDF-1ɑ in RCC by using the R packages “enrichplot”, “ggplot2”, and “circlize” and selecting the significantly enriched functional categories according to *p*-val < 0.05 and FDR q-val < 0.5. To explore the potential functional enrichment of SDF-1ɑ in RCC, we selected the significantly enriched functional categories based on *p*-val < 0.05 and FDR q-val < 0.5 to obtain the proportion and significance level of SDF-1ɑ in the BP, CC, and MF functional categories.

Download the c2.cp.kegg.v7.4.symbols.gmt file from the GSEA website and explore the potential signaling pathways of SDF-1ɑ in RCC through the R package “enrichplot”. The top 5 signaling pathways that were significantly enriched were selected based on the normalized enrichment score (NES), NOM *p* value < 0.05 and FDR q value < 0.05 and were divided into two groups according to the median expression of SDF-1ɑ, namely, the high-expression group and the low-expression group.

### Research objects and methods

Study subjects and research methods A total of 166 new RCC patients and 166 healthy individuals who attended or underwent physical examination at the Affiliated Hospital of Inner Mongolia Medical University and People’s Hospital from December 2017 to December 2021 were selected and paired according to the same sex, with an age difference of no more than 5 years. In addition, detailed information about the patients was obtained by reviewing the electronic cases and face-to-face interviews and completing the clinical data questionnaires. The study subjects signed an informed consent form. Improved multiple ligase detection reaction (iMLDR) was used to analyze the gene loci of the patients and controls^10^. Genotyping was performed on cases and controls.

### Ethical approval and participatory consent

The study was fully reviewed and approved by the Medical Ethics Committee of Inner Mongolia Medical University (YKD202102003) and was conducted in accordance with the Declaration of Helsinki. Informed consent was obtained from all participants after the nature of the research principles, including confidentiality and freedom of choice, were explained.

### Main enzymes and reagents

The whole-blood DNA rapid extraction kit was produced by Beijing Tiangen Technology Co., Ltd., tag DNA polymerase was produced by Qiagen, DNA marker was produced by New England Biolabs, deoxyribose hexoside triphosphate was produced by Gener-ay Biotech, and the primers were synthesized by Sangyo Bioengineering (Shanghai) Co. The primers were synthesized by Sangyo Bioengineering (Shanghai) Co. iMLDR Multiplex was used for typing by Tianhao Biotechnology (Shanghai) Co.

### Research methodology

Peripheral blood specimen collection and preservation All the study subjects fasted for 6–8 h. After fasting, 2 ml of venous EDTA was collected, and an inert separator gel was used to promote coagulation; after centrifugation and collection of serum and blood cells, the samples were frozen at − 80 °C.

Extraction of human genomic DNA In total, 100 μl of whole blood was taken, and DNA was extracted in strict accordance with the instructions of the kit. The DNA was quantified via a UV spectrophotometer and stored at – 80 ℃ for spare use.

Genotype detection The blood genomic DNA of the study subjects was extracted, polymerase chain reaction primers were designed, and iMLDR was used to genotype the SDF-1ɑ and XRCC1 gene loci.

### Statistical methods

The raw data were imported into EpiData 3.1 software using the principle of double entry, and a database was established after organization. SPSS 25.0 software was used to analyze the data. The *χ*^2^ test was used to analyze whether the genotype frequency distribution of individual loci in the population complied with the Hardy‒Weinberg (H–W) balance test, and the t test or *χ*^2^ test was used to compare the general conditions and lifestyle behavior of cases and controls according to the nature of the indexes. The multifactorial conditional logistic regression model was used to analyze the correlation between genetic polymorphisms and lifestyle habits and the risk of developing RCC. The correlation between gene polymorphisms and lifestyle behaviors and the risk of developing RCC was analyzed using a multifactorial conditional logistic regression model, and the interaction between genes and the environment was analyzed using the generalized multifactor dimensionality reduction (GMDR) method^11^. Generalized multifactor dimensional reduction (GMDR) was used to analyze the interaction between genes and the environment.

## Results

### Exploring the effect of SDF-1ɑ and XRCC1 expression on RCC based on raw letter analysis

#### Differences in the expression of SDF-1ɑ and XRCC1 in cancerous and normal tissues

**Figure 1:**
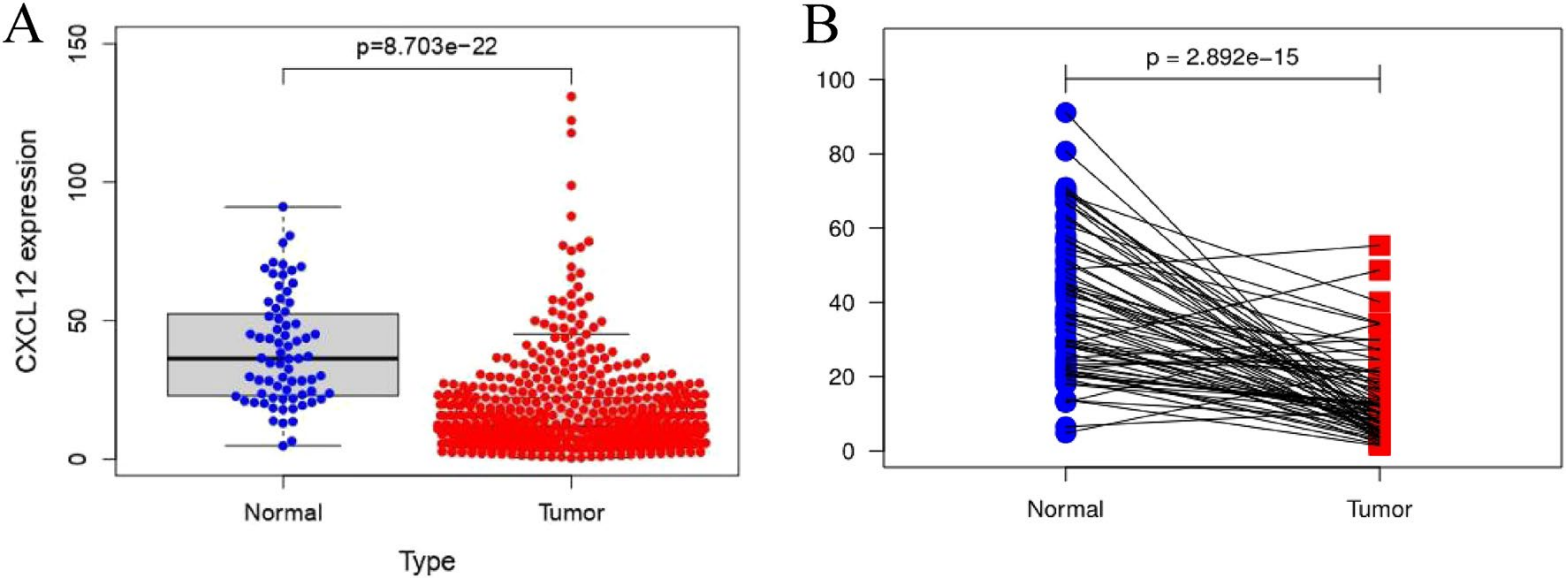
Expression of SDF-1ɑ (CXCL12) in RCC. (**A**) Expression of SDF-1ɑ (CXCL12) in cancerous tissue and normal tissue. (**B**) Expression of SDF-1ɑ (CXCL12) in paired tissue.

**Figure 2:**
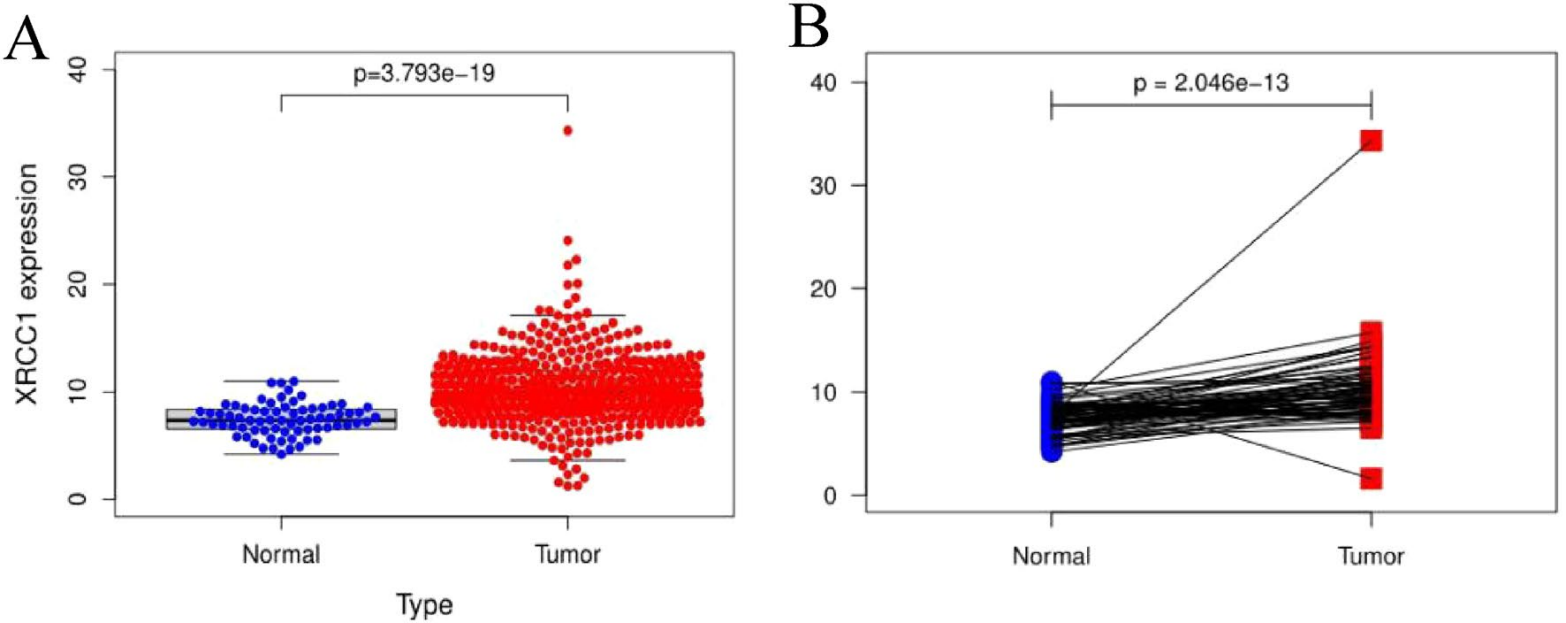
XRCC1 expression in RCC (**A**) XRCC1 expression in cancerous and normal tissues (**B**) XRCC1 expression in paired tissues.

The results showed that the expression of SDF-1ɑ (CXCL12) in normal tissues was significantly greater than that in cancerous tissues (Fig. 1A,B). The expression of XRCC1 in cancerous tissues was significantly greater than that in paracancerous tissues (*P* < 0.001) (Fig. 2A,B).

#### Survival analysis

**Figure 3:**
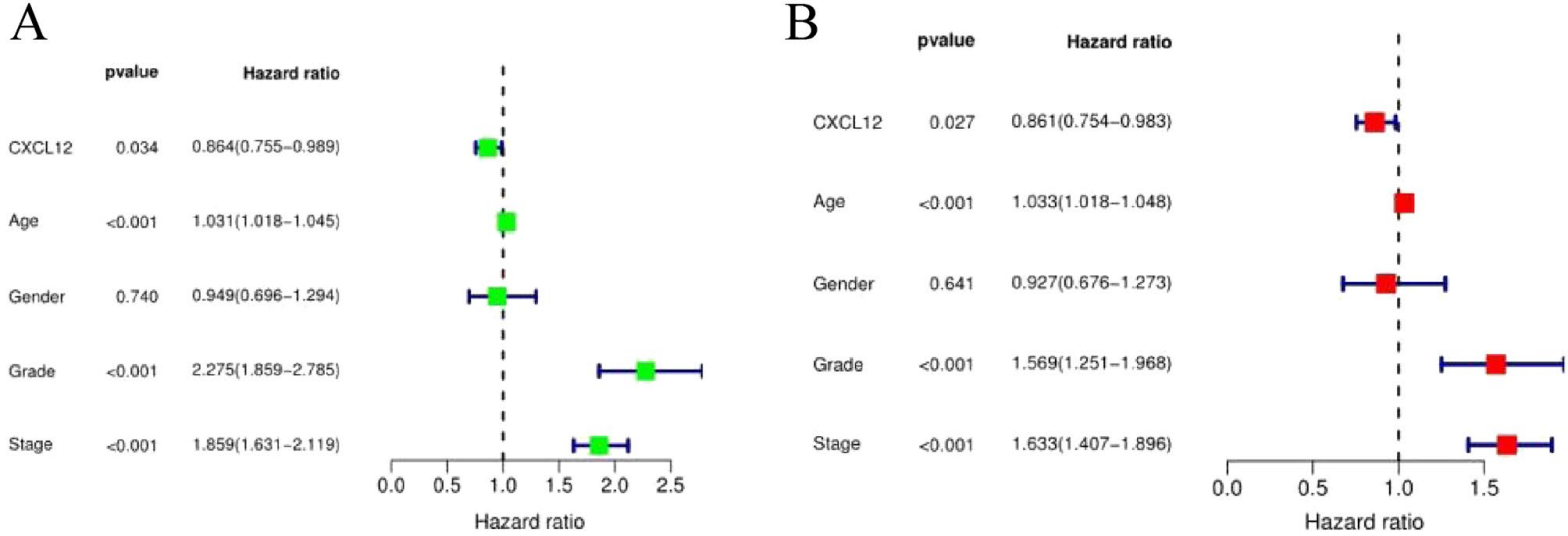
Relationships between SDF-1ɑ (CXCL12) expression and overall survival (**A**) One-way Cox regression analysis (**B**) Multifactor Cox regression analysis.

Survival analysis was performed using kidney cancer samples from the TCGA public database. Univariate Cox analysis revealed that SDF-1ɑ (CXCL12) expression, age, histologic grade, and clinical stage were associated with overall survival in renal cancer patients, and subsequent multivariate Cox analysis revealed that age, histologic grade, and clinical stage were independent risk factors for overall survival in renal cancer patients; moreover, high SDF-1ɑ (CXCL12) expression was a protective factor (Fig. 3A,B).

#### GO enrichment analysis

**Figure 4:**
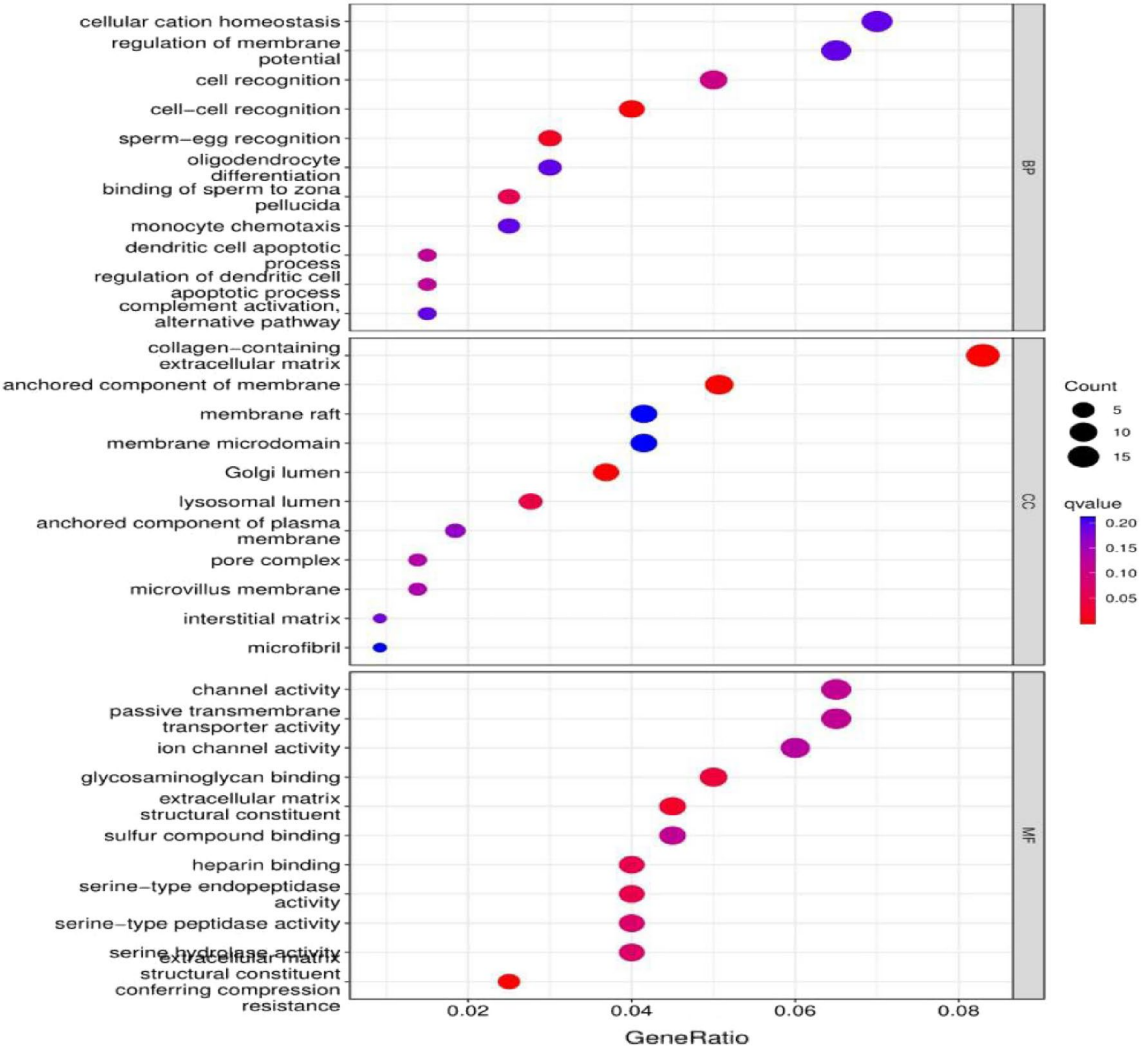
GO function set analysis of the SDF-1ɑ (CXCL12) gene.

GO enrichment analysis was performed on the SDF-1ɑ (CXCL12) gene, and the relevant biological processes were plotted as bubble plots according to the degree of enrichment (Fig. 4). GO enrichment analysis of BP was mainly focused on cellular cation homeostasis, regulation of membrane potential, cell recognition, cell‒cell recognition, and sperm-egg recognition. CC enrichment was mainly focused on collagen-containing extracellular matrix, anchored component of membrane, membrane raft, membrane microdomain, and Golgi lumen. MF enrichment was mainly related to channel activity and passive transmembrane transporter activity, ion channel activity, glycosaminoglycan binding, and extracellular matrix structural constituents.

#### GSEA enrichment analysis

**Figure 5:**
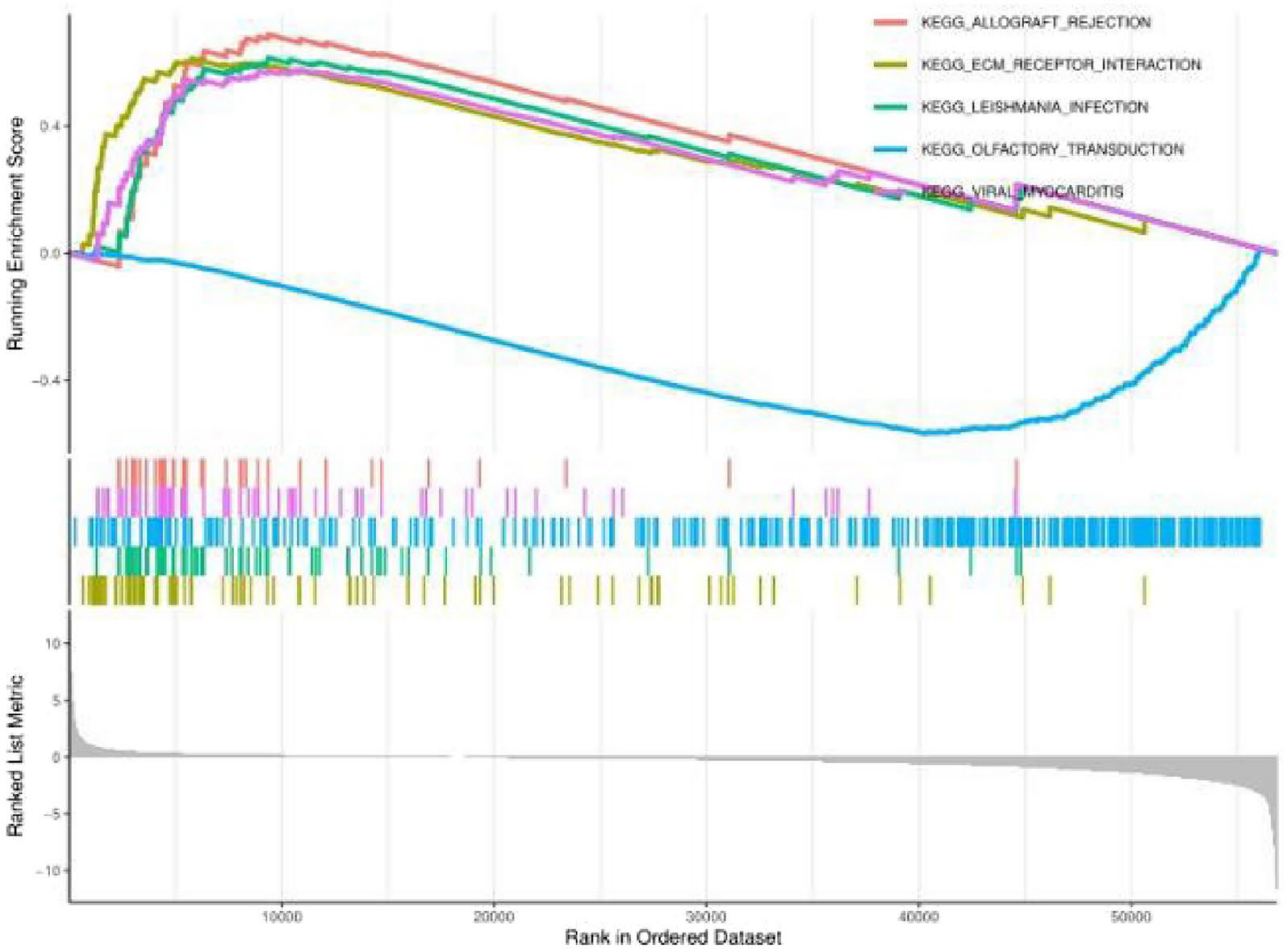
GSEA enrichment map of the SDF-1ɑ (CXCL12) gene.

GSEA of high and low SDF-1ɑ(CXCL12) expression data sets revealed that allograft rejection, ECM receptor interaction, Leishmania infection, Leishmania infection, and viral myocarditis in SDF-1ɑ(CXCL12) was significantly enriched in the high-expression group (Fig. 5).

### Comparison of general information between the case and control groups

Smoking history, drinking history, education level, pesticide exposure history, food saltiness, fruit intake, meat and vegetable combinations, type of cooking oil, milk consumption, type of drinking water, hair dyeing, labor intensity, and urine holding were significantly different between the two groups (*p* < 0.05); BMI and ethnicity were not significantly different between the two groups (*p* > 0.05), as shown in Tables 1 and 2.

**Table 1:** Comparison of demographic data between the two groups (x ± s).

Indicators	Cases (n = 166)	Control (n = 166)	*χ*^2^	*P*
BMI	24.07 ± 3.39	23.91 ± 2.78	0.446	0.656
Ethnicity			3.634	0.057
Han	149	137		
Other	17	29		
Education level			30.101	< 0.001
Elementary school and below Junior high school	149	107		
High school or junior college College and above	17	59

**Table 2:** Comparison of life history data between the two groups (x ± s).

Indicators	Cases (n = 166)	Control (n = 166)	*χ*^2^	*P*
Smoking			5.315	0.021
Yes	95	74		
No	71	92		
Drinking			6.119	0.013
Yes	76	54		
No	90	112		
Pesticide exposure			13.620	< 0.001
Yes	40	15		
No	126	151		
Food salinity			7.143	0.008
Salty/light	109	85		
Moderate	57	81		
Fruit intake			8.900	0.003
Frequently	79	106		
Occasionally/not	87	60		
Meat and vegetables			21.405	< 0.001
Balanced meat and vegetables	69	111		
Meat-based/vegetarian-based	97	55		
Edible oil consumption			34.772	< 0.001
Animal oil	52	11		
Vegetable oil	107	138		
Other	7	17		
Drinking milk			5.658	0.017
Frequently	52	73		
Occasionally/not	114	93		
Type of drinking water			15.319	< 0.001
Running water	100	73		
Deep well water	25	18		
Pure water	41	75		
Hair dye			4.476	0.029
Yes	68	49		
No	98	117		
Exercise			5.996	0.014
Moderate	30	49		
Stronger/lighter	136	117		
Hold your urine			6.566	0.010
Yes	13	3		
No	153	163		

### Hardy‒Weinberg equilibrium test

Allele frequencies and genotype frequencies in the population did not change with each generation. The rs1801157 genotype of the SDF-1ɑ gene and the rs1799782 genotype of the XRCC1 gene obeyed the H-W equilibrium test (*P* > 0. 05) in this study, indicating that the samples could better replace the population and conformed to the law of genetic equilibrium of the population, as shown in Tables 3 and 4.

**Table 3:** Hardy‒Weinberg equilibrium test for the SDF-1ɑ gene polymorphism at the rs1801157 locus.

Genotypes	Cases	Control	*χ*^2^	*P*
CC	91	94	0.855	0.652
CT	68	62		
TT	7	10

**Table 4:** Hardy‒Weinberg equilibrium test for the rs1799782 polymorphism locus of the XRCCI gene.

Genotypes	Cases	Control	*χ*^2^	*P*
AA	11	14	0.668	0.716
GA	74	68		
GG	81	84		

### Relationships between polymorphic SDF-1ɑ and XRCC1 gene loci and clinicopathologic features of RCC

According to the WHO^12^ pathohistological types of RCC, patients with RCC were grouped according to different categories, including clear cell renal tumors, papillary renal tumors, oncocytic and chromophobe renal tumors, and molecularly defined renal carcinomas. After sorting the collected samples, we found that the samples of collected ductal tumors, other kidney tumors and molecularly defined kidney cancer accounted for a small proportion of the samples; therefore, we unified these three categories into other tumors, and the distributions of alleles and genotypes of each locus were compared with each other among the subgroups with or without differences. We classified the pathological sections of the study subjects into histological and TNM grades according to WHO standards and compared the differences between them. The results showed that the rs1799782 genotype (G:A) of the XRCC1 gene was significantly different between different pathohistological types of RCC (*p* < 0.05), whereas no statistically significant difference was found between the rs1801157 genotype of the SDF-1ɑ gene and clinicopathological features, as shown in Tables 5 and 6.

**Table 5:** Allele/genotype frequencies of the rs17997821 locus of the XRCC1 gene vs. Relationships between clinicopathologic features of RCC.

	Equivalent position Genes		*X*^2^	*P*		Genotypes		*X*^2^	*P*
Indicators	A	G			A/A	G/A	G/G		
Histopathologic type			4.127	0.243				11.806	0.044
Clear cell renal tumors	57	139			9	39	50		
Papillary renal tumors	7	15			3	1	7		
Oncocytic and chromophobe renal tumors	33	71			4	25	23		
Other renal tumors	6	4			2	2	1		
Histologic grade (G)			2.878	0.390				4.788	0.618
G1	58	10			7	20	19		
G2	131	45			10	37	53		
G3	22	16			1	9	9		
G4	1	1			0	1	0		
Clinical staging			3.947	0.263				9.939	0.085
I	85	175			13	59	58		
II	15	33			4	7	13		
III	3	19			1	1	9		
IV	0	2			0	0	1		
Original (primary) tumor			3.037	0.375				7.740	0.189
T1	87	191			13	61	65		
T2	10	18			3	4	7		
T3	3	17			1	1	8		
T4	1	5			0	1	2		
Regional lymph nodes			3.351	0.318				1.914	0.780
NX	1	5			0	1	2		
N0	101	215			18	65	75		
N1	1	9			0	1	4		
Distant metastases			1.462	0.493				2.540	0.724
MX	2	4			0	2	1		
M0	101	221			18	65	78		
M1	0	4			0	0	2

**Table 6:** Allele/genotype frequencies of the rs1801157 locus of the SDF-1ɑ gene vs. Relationships between clinicopathologic features of RCC.

	Equivalent position Genes		*X*^2^	*P*		Genotypes			*X*^2^	*P*
Indicators	C	T			C/C	C/T		T/T		
Histopathologic type			1.220	0.751					1.557	0.967
Clear cell renal tumors	152	44			58	36	4			
Papillary renal tumors	19	3			8	3	0			
Oncocytic and chromophobe renal tumors	82	22			32	18	2			
Other renal tumors	9	1			4	1	0			
Histologic grade (G)			2.114	0.526					4.534	0.763
G1	77	15			32	13	1			
G2	154	46			58	38	4			
G3	29	9			11	7	1			
G4	2	0			1	0	0			
Clinical staging			4.200	0.211					8.207	0.216
I	205	55			79	47	4			
II	41	7			18	5	1			
III	15	7			5	5	1			
IV	1	1			0	1	0			
Original (primary) tumor			1.241	0.775					5.198	0.482
T1	219	59			85	49	5			
T2	23	5			10	3	1			
T3	14	6			4	6	0			
T4	5	1			2	1	0			
Regional lymph nodes			3.259	0.187					5.854	0.231
NX	6	0			3	0	0			
N0	250	66			97	56	5			
N1	6	4			2	2	1			
Distant metastases			0.394	1.000					2.361	1.000
MX	5	1			2	1	0			
M0	254	68			99	56	6			
M1	3	1			1	1	0			

### Association of SDF-1ɑ and XRCC1 gene expression and lifestyle habits with kidney cancer

With the presence of RCC as the dependent variable, the rs17997821 locus of the XRCC1 gene, the rs1801157 locus of the SDF-1ɑ gene, smoking status, literacy level, holding urine, dyeing hair, exposure to pesticides, type of meat–vegetable combination, and labor intensity as the independent variables, the conditional logistic regression model was applied to investigate the associations between the polymorphisms of the SDF-1ɑ and XRCC1 genes and between lifestyle habits and RCC. Smoking, holding urine, and dyeing hair were risk factors for RCC, and regular intake of fruit was a protective factor against RCC, as shown in Table 7.

**Table 7:** Results of the RCC multifactor conditional logistic regression analysis model analysis.

Variant	OR	95% CI	*P*
rs1799782			
A/A	1		
G/A	1.636	(0.727,3.682)	0.234
G/G	0.905	(0.576,1.422)	0.666
rs1801157			
C/C	1		
C/T	1.308	(0.424,4.034)	0.641
T/T	0.995	(0.317,3.128)	0.993
Smoking			
Yes	1		
No	1.852	(1.105, 3.103)	< 0.001
Education level			
Elementary school and below Junior high school	1		
High school or junior college College and above	4.965	(2.503, 9.846)	< 0.001
Hold your urine			
Yes	1		
No	4.483	(1.094, 18.373)	0.037
Hair dye			
Yes	1		
No	1.948	(1.135, 3.342)	0.016
Pesticide exposure			
Yes	1		
No	3.105	(1.487, 6.481)	0.003
Meat and vegetables			
Balanced meat and vegetables	1		
Meat-based/Vegetarian-based	2.418	(1.463, 3.997)	0.001
Exercise			
Moderate	1		
Stronger/lighter	2.113	(1.159, 3.854)	0.015

### Gene‒environment interactions and the risk of developing RCC

Using the GMDR method^13^, environmental factors were incorporated into the model for analysis, and the results showed that the rs1799782 locus of the XRCC1 gene in RCC interacted with smoking, alcohol consumption, pesticide exposure, hair dyeing, and urine holding, with a cross-consistency of 100%, an accuracy of 0.6854 in the training samples, and an accuracy of 0.6276 in the test samples, with *P* = 0.0010, suggesting that rs1799782 interacted with smoking, alcohol consumption, pesticide exposure, hair coloring, and urine holding had an interaction effect; i.e., the coexistence of these five factors could increase the risk of RCC by 2.94 times (OR = 2.94, 95% CI 1.23–7.02) (Table 8).

**Table 8:** GMDR analysis of RCC gene‒environment interaction models.

Model	Training Bal	Testing Bal	Sign Test(*p*)	CV Consistency
C	0.5771	0.5296	0.1719	7/10
C D	0.6175	0.6176	0.0107	10/10
C D E	0.6332	0.6162	0.0107	9/10
B C D E	0.6466	0.5820	0.0547	6/10
A B C E rs1799782	0.6673	0.5942	0.0010	8/10
A B C D E rs1799782	0.6854	0.6276	0.0010	10/10

A Smoking B Drinking alcohol C Pesticide exposure D Dyeing hair E Holding urine.

## Discussion

According to data provided by the World Health Organization, there are more than 140,000 RCC-related deaths each year, and RCC is the 13th most common cause of cancer death worldwide^14^. Although early diagnosis of RCC is critical for treating patients and reducing mortality, optimal screening modalities and methods have not been established^1^; therefore, exploring the underlying molecular mechanisms of RCC and identifying biomarkers associated with early diagnosis, treatment, and prognosis are important for guiding clinically individualized treatment regimens.

Stromal cell-derived factor-1 (SDF-1ɑ), also known as chemokine CXCL12 (C-X-C chemokine ligand 12), is a class of cytokines with chemotactic activity. Moreover, targeting the CXCL12-mediated axis can control tumors and the tumor microenvironment by playing dual roles inanti-tumor^15^, Zhou^16^ demonstrated that the CXCL12/CXCR4 axis can be used as a molecular target for cancer therapy. Human X-ray cross-complementary repair gene 1 (XRCC1) is an important DNA damage repair gene and is one of the key genes in the base excision repair (BER) pathway. Polymorphisms in the XRCC1 gene have been reported to be associated with susceptibility to a variety of cancers^17–22^. Akhmadishina et al.^22^ found that the XRCC1 gene polymorphism c.839 G > A may promote early and advanced renal cancer. Therefore, this study analyzed and explored the relationship between genetic polymorphisms and RCC susceptibility from a genetic point of view to provide scientific biomarkers for the effective prevention of RCC and analyzed and studied the associations of SDF-1ɑ and XRCC1 gene polymorphisms with lifestyle habits in RCC to provide a scientific basis for the effective prevention of RCC development.

In this study, by analyzing the differences in the expression of SDF-1ɑ and XRCC1 between tumor tissues and normal tissues, we found that the expression of SDF-1ɑ was increased in normal tissues and that the expression of XRCC1 was increased in RCC tissues. Analysis of the survival data of RCC patients in the TCGA database showed that high SDF-1ɑ expression may play a role in the treatment of RCC and may have a positive antitumor immune effect. SDF-1ɑ has been shown to play a protective role against acute kidney injury (e.g., ischemia/reperfusion injury)^23^. A study by Armstrong et al.^24^ showed that SDF-1ɑ was the only vascular factor reduced by low-risk disease and that SDF-1ɑ was associated with improved survival, which is consistent with our study.

GO and GSEA of SDF-1ɑ revealed that the genes enriched in the GO enrichment analysis were involved mainly in channel activity, passive transmembrane transporter protein activity, ion channel activity, glycosaminoglycan binding, and structural components of the extracellular matrix, while the genes enriched in the GSEA were involved mainly in allografts, extracellular matrix receptor action, and the leishmaniasis pathway. Numerous studies have shown that extracellular matrix elasticity or stiffness affects fundamental cellular processes, including cell spreading, growth, proliferation, migration, differentiation and organoid formation^25^. Najafi et al. stated that cancer cell proliferation, migration and invasion, as well as angiogenesis, are consequences of the stiffness and degradation of the extracellular matrix and that establishing homeostasis in the extracellular matrix (ECM) to facilitate the penetration of chemotherapeutic agents and improve the efficacy of antitumor therapies would be a promising strategy^26^. In his review, Anderson showed that ion channels are important for the regulation of tissues and cancer stem cells and that the effects of ion channels on cancer cellular processes lead to cancers described as channelopathies^27^. However, there are no studies on the association between SDF-1ɑ and the RCC signaling pathway. Our results suggest that SDF-1ɑ may influence the progression of RCC by modulating multiple signaling pathways.

In the present study, smoking, urine holding and hair coloring were found to be risk factors for RCC, and regular intake of fruits was found to be a protective factor against RCC. Macleod LC found an independent correlation between smoking and RCC in the VITAL study^28^. Similarly, Lotan Y et al. in the PLCO trial, confirmed that smoking intensity was significantly associated with a high risk of RCC^29^, the present study found that the risk of RCC in smokers was 1.85 times higher than that in nonsmokers by Logistic analysis (OR = 1.852; 95% CI 1.105, 3.103), which is in line with the results of previous studies. The present study showed that there was no statistically significant correlation between alcohol consumption and susceptibility to RCC (all *P* > 0. 05), but moderate alcohol consumption has been shown to have a protective effect on the incidence of RCC^30,31^. However, these findings are not consistent with our findings, and we believe that this may be due to sampling errors or the insufficient number and type of samples analyzed in this study. These limitations resulted in a lack of statistically significant associations between alcohol consumption and susceptibility to RCC and may need to be further verified in a study with a larger sample size.

The genetic polymorphisms of the rs1799782 locus of the XRCC1 gene and the rs1801157 locus of the SDF-1ɑ gene showed that patients with each genotype had fewer lymph node metastases and distant metastases; the clinical stages were mostly stage I and II; and the pathohistological type was mostly clear-cell renal cell carcinoma. Although the rs1799782 locus of the XRCC1 gene was detected, the rs1801157 locus of the SDF-1ɑ gene and genotype were not correlated with tumor location or stage. Although the rs1799782 locus of the XRCC1 gene, the rs1801157 locus of the SDF-1ɑ gene allele and the genotype were not significantly correlated with tumor location or stage, the difference in rs1799782 of the XRCC1 gene among the different pathohistological types may be key factors in the onset and pathological development of renal cell carcinoma.

Multifactor dimensionality reduction (MDR) is a nonparametric, higher-order interaction analysis method developed in recent years without the need for a genetic model, while the extension method generalized multifactor dimensionality reduction (GMDR), based on the basic principles of MDR, makes it possible not only to analyze continuous variables but also to include covariates, thus controlling the interference caused by covariates and improving the accuracy of prediction^32^. The GMDR model enables not only the analysis of continuous variables but also the incorporation of covariates, thus controlling the interference caused by covariates and improving the accuracy of prediction^13^. In this study, the GMDR method was used to analyze the interaction between genes and the environment. The results showed that the rs1799782 locus of the XRCC1 gene in RCC interacted with smoking, alcohol consumption, pesticide exposure, hair dyeing, and urine holding.

In summary, although this study has several limitations, such as a small sample size and admission rate bias due to the two-way selectivity between patients and hospitals, which results in cases and controls not being a random sample of the whole population, and the lack of basic validation experiments on the SDF-1ɑ and XRCC1 genes, this study confirmed the downregulation of SDF-1ɑ expression and upregulation of XRCC1 expression in kidney cancer tissues by bioinformatic methods and integrated information from the TCGA database. However, this study confirmed that SDF-1ɑ expression was downregulated and that XRCC1 expression was upregulated in renal cancer tissues by bioinformatics methods and integrated information from the TCGA database. High SDF-1ɑ expression may be a protective factor for overall survival in renal cancer patients, providing a basis for clinical experimental studies. In future studies, we hope to further expand the sample size of clinical studies and further conduct basic studies in vivo and in vitro to further corroborate the possible molecular mechanism of the SDF-1ɑ gene in the development of renal cancer.

### Supplementary Information

Supplementary Information 1.

Supplementary Information 2.

## Data Availability

The data generated or analyzed during this study are included in this published article and its supplementary information files. Publicly available datasets were analyzed in this study. These data can be found in the Cancer Genome Atlas (https://portal.gdc.cancer.gov/). Due to the privacy of the research subjects, lifestyle data cannot be publicly obtained.
