# Supplementary material for: Correlations of SDF-1ɑ and XRCC1 gene polymorphisms with the risk of renal cancer development and bioinformatics studies of SDF-1α and XRCC1 and the prognosis of renal cancer
Source: Sci Rep. 2024 Feb 9;14:3367. doi: 10.1038/s41598-024-53808-4 (PMC10858090; doi:10.1038/s41598-024-53808-4)
Supplement: Supplementary file 1 — Supplementary Information 1. [file 41598_2024_53808_MOESM1_ESM.pdf]

Correlation of SDF-1α and XRCC1 genes polymorphisms with the risk of renal cancer development and bioinformatics study of SDF-1α and XRCC1 and prognosis of renal cancer

Wenjing Zhang1、Yubo Su1、Genquan Yue2 、Lingyan Zhao1,3 、Hailing Li1,3、Min Jia1、Yuqi Wang1、Dongyang Liu1 、Haisheng Wang1,4,*、Yumin Gao1,3,*

1School of Public Health, Inner Mongolia Medical University, Hohhot, China.
2Department of Urology, Affiliated Hospital of Inner Mongolia Medical University, Hohhot, China.
3Key Laboratory of Molecular Epidemiology of Chronic Diseases, Inner Mongolia Medical University, Hohhot, China.
4Department of Biochemistry and Molecular Biology,School of Basic Medicine, Inner Mongolia Medical University, Hohhot, China.
*Correspondence: yxywhs001@126.com; gaoyumin0712@163.com

Living Conditions and Health Questionnaire

No. Name:

Classification: 1. Cases 2. Controls

Hello:

I am a health surveyor from Inner Mongolia Medical University. We are now working with Inner Mongolia Affiliated Hospital, Inner Mongolia People's Hospital and other related organizations to conduct a survey on the risk factors for urological diseases, including your past health, diet and other lifestyle habits. All survey information is confidential and your name or other information that identifies you will not appear on any of the survey reports.

informed consent

I have understood the description of this health survey and am satisfied with the investigator's presentation, and I voluntarily participate in the

Add this inquiry.

Signature of survey respondent:

Date of signature:: Month and year

Investigator's statement

I have fully explained the matters to the respondents and the respondents have sufficient understanding of the study.

Investigators:

Date of signature

A. General

A01 Sex: 1. male 2. female Oral

A02 Date of Birth Month of Year (age) Mouth

A03 Ethnic Han: 1. Han 2. Hui 3. Mongolian 4. Other Populations

A04 Place of birth: province, city (county)

Place of origin: Province, city (county)

A05 Height: Weight: BMI.

A06 Occupation: 1. total mental labor 2. major mental labor 3. major physical labor 4. total physical labor Mouth

A07 Literacy level: 1. Elementary school or below 2. Junior high school 3. High school/secondary school 4. College or above Mouth

A08 Marital status: 1. unmarried 2. married 3. cohabiting 4. divorced 5. widowed 6. separated 口

A09 Family size: persons

A10 Family Address Province, City, County (District) Township (Street) Village (No.) Group (Block) No.

Contact phone number: A11 Current workplace:

Case-related information

A14 Hospitalization number A15 Pathology number

A16 Pathologic type: 1. clear cell type 2. papillary renal cell carcinoma 3. smoky renal cell carcinoma 4. others

A17 Pathologic classification: 1. Grade I 2. Grade II 3. Grade III 4. Other Oral

A18 Clinical staging: 1. Stage 1 2. Stage 2 3. Stage 3 Oral

A18a Primary tumor staging: 1.Tx 2.To 3.T1 4.T2 5.T3 6.T4

A18b Regional lymph node juvenile staging: 1.Nx 2.N0 3.N1 4.N2 5.N3

A18c Distant metastasis staging: 1.Mx 2.M0 3.M1

Comparison of relevant information

A19 hospitalization number

A20 Outpatient number

A21 Diagnosis of diseases

A22 Comparison of departments attended

B. **Occupational and past medical history**

B01 Are you regularly exposed to pesticides or insecticides? 1. Yes 2. No Mouth

Such as regular exposure to pesticides or insecticides:

B011 Exposure to pesticides by: 1. spraying 2. seed dipping 3. selling 4. other □

B012 Dermal contamination during exposure to pesticides: 1. none 2. small amount 3. large amount Oral

B02 Have you ever worked in a factory? 1. Yes 2. No

B021 If you have worked in a factory, which of the following types of factories was it?

1.Dye factory 2.Electroplating factory 3.Rubber factory 4.Chemical factory 5.Leather factory 6.Paint factory 7.Coating factory 8.Other □

The main products produced in the factory are:

B022 Your occupational exposure:
labor

	job category	years of experience	What are the main toxic or hazardous elements to which you are exposed?

B03 Have any of your coworkers ever had a urological disorder? 1. Yes 2. No 3. Unknown Mouth

B04 Do you have a history of chronic hepatitis, cirrhosis, or long-term poor liver function? 1. yes 2. no Mouth

B05 Do you have any other urinary diseases: 1. Prostate disease 2. Bladder disease 3. Other Mouth

B06 Do you have high blood pressure: 1. Yes 2. No Mouth

If yes, what kind of medications are taken: 1. diuretics 2. beta-blockers 3. calcium channel blockers 4. angiotensin-converting enzyme inhibitors 5. angiotensin di-receptor inhibitors Specific drug names: oral

B07 Do you have diabetes: 1. Yes 2. No Mouth

C Living habits and environmental conditions

Smoking

C01 Do you smoke ("Smoking" means at least one cigarette a day for more than 6 months)

1. Yes, still smoke 2. Yes, but have quit smoking 3.

If smoking: How old did C011 start smoking regularly? Age, how many years did he smoke? Years

C012 On average, how many cigarettes per day do you smoke when you smoke regularly? Sticks/day

C013 Have you ever quit smoking (not smoked for more than three months) 1. yes 2. no Mouth

C014 If you have quit smoking, how many years have you quit smoking? Years

Passive smoking

C02 Do family members living with you smoke? 1. yes 2. no Mouth

C021 If you smoke, how many cigarettes per day on average? Sticks/day

C022 How many years did you live together while he/she smoked? Years.

C03 At your workplace, are you exposed to others smoking? 1. Yes 2. No Mouth

C031 If you were exposed to someone else's smoking: Total years of exposure Years

What is the level of exposure to C032? 1. light 2. moderate 3. heavy Mouth

Alcohol consumption

C04 Do you drink alcohol ("Drinking" means at least once a week for more than one year)?

1. Yes, still drinking 2. Yes, but have stopped drinking 3.

C041 If you drink alcohol, how old were you when you started drinking? Age, total number of years of drinking? Years

C042 Have you ever stopped drinking? 1. yes 2. no Mouth

C043 If ever sober, at what age did you start drinking? Years How many years of sobriety? How many years?

C044 Type and amount of alcohol you drink most often: 1 White wine (38° or more) pounds per week

2. Wine kg per week 3. Beer bottle per day 4. Other kg per week Mouth

Diet

C05 In the last 5 years, how many meals a day do you usually have? Meal Mouth

C06 In the last five years, the food you ate was often: 1. rather salty 2. moderate 3.

bland Mouth

C07 Do you often eat fruits? 1. every day 2. often (3-5 days) 3. occasionally (1-2) 4. not at all

mouth

C08 The combination of meat and vegetables in your daily diet: 1. Meat-based 2. Balanced meat and vegetables 3. Vegetarian-based 口

C09 What kind of food oil do you usually use in cooking? 1. animal oil 2. vegetable oil 3. other 口

C10 Do you drink milk regularly? 1. every day 2. often (3-5) 3. occasionally (1-2) 4. not quoted 口

C11 How often do you eat barbecue? 1. often (once a month) 2. occasionally (once every six months) 3. not at all Mouth

C12 What kind of water do you mainly drink? 1. tap water 2. deep well water 3. purified water 口

C13 Your daily water intake is about 1.500ml or less 2.500-1000ml 3.1000ml-3000ml

4.3000+ Mouth

D Other habits and environmental conditions

D1 Do you color your hair? 1. Yes 2. No Mouth

D11 If you color your hair, how often do you usually color your hair? 1. one month 2. one month 3. six months to one year
4. More than one year Oral

D2 Your usual physical labor intensity 1. strong 2. moderate 3. light Mouth

D3 What is your usual level of psychological tension psychological stress? 1. relaxed 2. not too nervous 3. more nervous
4. Nervous, stressed out, mouth.

D4 Do you exercise regularly? 1. exercise every day 2. often (3-5 days) 3 occasionally (1-2 days)

4. Not at all Mouth

D5 Do you have the habit of holding your urine? 1. often 2. occasionally 3. not at all Mouth

D6 Do you use air fresheners? 1. every day 2. often (3-5 days) 3. occasionally (1-2 days)

4. No Mouth

D7 The main fuel used in your household for heating and cooking: 1. natural gas 2. gas 3. firewood 4. coal 口

E Family history

E1 Has anyone in your immediate family, parents, siblings, or children, ever had a tumor?

1. Yes 2. No 3. Unknown Mouth

If so, what are the specific tumors:

E2 Has anyone in your immediate family, parents, siblings, or children, ever had a urological disease?

1. Yes 2. No 3. Unknown Mouth

If so, what exactly:

E3 Whether any of your other family members grandparents, uncles, aunts, or uncles have had a tumor

1. Yes 2. No 3. Unknown Mouth

If so, what are the specific tumors:

E4 Have any of your other family members grandparents, uncles, aunts, uncles, or uncles suffered from urological disease?

1. Yes 2. No 3. Unknown Mouth

If so, what exactly:

Investigator's Postscript

Cooperation of survey respondents: 1. very good 2. good 3. fair 4. poor

Is the respondent credible in answering the question? 1. Credible 2. Fairly credible

Interview location: 1. on the hospital 2. at work 3. at the respondent's home 4. at other locations

Evaluation of the quality of the survey material as a whole: 1. high 2. average 3. less satisfactory

Site: 1. kidney 2. bladder 3. prostate 4. other Blood: 1. retained 2. not retained

Tissue samples: 1. retained 2. not retained

Date of investigation Month of year
inspector
